# Rapamycin Upregulates Autophagy by Inhibiting the mTOR-ULK1 Pathway, Resulting in Reduced Podocyte Injury

**DOI:** 10.1371/journal.pone.0063799

**Published:** 2013-05-08

**Authors:** Lingling Wu, Zhe Feng, Shaoyuan Cui, Kai Hou, Li Tang, Jianhui Zhou, Guangyan Cai, Yuansheng Xie, Quan Hong, Bo Fu, Xiangmei Chen

**Affiliations:** 1 State Key Laboratory of Kidney Diseases, Department of Nephrology, PLA General Hospital and Military Medical Postgraduate College, Beijing, China; 2 Medical College, NanKai University, Tianjin, China; University of Houston, United States of America

## Abstract

The podocyte functions as a glomerular filtration barrier. Autophagy of postmitotic cells is an important protective mechanism that is essential for maintaining the homeostasis of podocytes. Exploring an in vivo rat model of passive Heymann nephritis and an in vitro model of puromycin amino nucleotide (PAN)-cultured podocytes, we examined the specific mechanisms underlying changing autophagy levels and podocyte injury. In the passive Heymann nephritis model rats, the mammalian target-of-rapamycin (mTOR) levels were upregulated in injured podocytes while autophagy was inhibited. In PAN-treated podocytes, mTOR lowered the level of autophagy through the mTOR-ULK1 pathway resulting in damaged podocytes. Rapamycin treatment of these cells reduced podocyte injury by raising the levels of autophagy. These in vivo and in vitro experiments demonstrate that podocyte injury is associated with changes in autophagy levels, and that rapamycin can reduce podocyte injury by increasing autophagy levels via inhibition of the mTOR-ULK1 pathway. These results provide an important theoretical basis for future treatment of diseases involving podocyte injury.

## Introduction

Podocytes, also called visceral glomerular epithelial cells, have a unique and complex structure, and are involved in the glomerular capillary loop filtration barrier. Podocytes are injured in many forms of human glomerular disease, such as minimal change disease (MCD), focal segmental glomerulosclerosis (FSGS), diabetic nephropathy, membranous glomerulopathy, lupus nephritis, and experimental glomerular disease. Therefore, understanding podocyte injury and repair mechanisms is of great significance to the prevention and treatment of kidney disease. [1], [2]

Podocytes are similar to neurons in that they are terminally differentiated cells and have extremely limited proliferative capacity. When injury and loss occur, podocytes cannot proliferate to cope with the adverse living environment, and thus the podocyte response mechanism must adjust to outside stress for survival [3]. Cell autophagy is a process that transports damaged cells, degenerated or aging proteins, and organelles to the lysosomes for digestion and degradation. It effectively copes with external environmental changes, resists exogenous stimulation, and maintains homeostasis. At the same time, autophagy serves as a defense mechanism by clearing damaged cytoplasmic organelles [4]. Thus, its presence is essential for long-lived terminally differentiated cells. An earlier study [5] demonstrated that the autophagy level was much higher in podocytes than in other renal cells, and podocytes depend on autophagy to maintain homeostasis. A more recent study [6] using podocyte-specific deleted Atg5 gene transgenic mice eloquently demonstrated the importance of autophagy to podocytes. Under pathophysiological conditions, the loss of autophagy in podocytes seems to result in a dramatically increased susceptibility to glomerular disease, uncovering the particular importance of autophagy as a critical homeostatic mechanism for maintaining podocyte integrity not only under physiological but also under stress conditions.

Mammalian target-of-rapamycin (mTOR) is a recently discovered evolutionarily conserved protein kinase that is also an important signal transduction molecule. It senses environmental amino acids, ATP, growth factors, and insulin levels, and has an important regulatory role in cell growth. mTOR exists in two complexes, termed mTOR complex-1 (mTORC1) and mTORC2. mTORC1 is mainly involved in protein synthesis and cellular energy metabolism processes and is inhibited by rapamycin. mTORC2 plays an important role in the regulation of the cytoskeleton and is insensitive to rapamycin [7], [8], [9]. Under basal conditions, the glomerulus seems to be maintained by extremely low mTOR, whereas abnormal expression of mTOR is found in kidney lesions [3]. Previous experimental studies in diabetic nephropathy and crescentic nephropathy animal models have found that mTOR is upregulated in podocytes and damaged podocytes [10], [11], [12]. Recent studies have found that mTOR inhibits autophagy by producing ULK1, a member of the autophagy initiation associated complex ULK1-ATG13-FIP200, resulting in loss of its kinase activity by phosphorylating Ser757, and participating in a variety of pathological and physiological processes [13], [14], [15], [16], [17], [18], [19], [20]. Rapamycin, also known as sirolimus, is a natural antibiotic. By binding to FK506-binding protein of 12 kDa (FKBP12), it is an acute specific inhibitor of mTORC1. Rapamycin is essential not only for the identification of mTOR, but also for elucidating mTOR-dependent signaling events and their role in metabolism and disease [21], [22], [23]. Therefore, the use of this immunosuppressing drug for inhibiting the mTOR-ULK1 pathway may serve as a new basis for the treatment of podocyte injury in renal disease.

In the present study, using an *in vivo* rat model of passive Heymann nephritis and *in vitro* puromycin amino nucleotide (PAN)-cultured conditionally immortalized mouse podocyte cells, we found that mTOR destroyed podocyte homeostasis and induced podocyte injury through the mTOR-ULK1 pathway, resulting in the downregulation of autophagy. Rapamycin pretreatment inhibited the activation of the mTOR-ULK1 pathway, thus increasing the level of autophagy and reducing podocyte injury. However, treatment of podocytes with RNAi-mediated siRNA interference for ATG7 (a key protein in autophagy) did not reduce podocyte injury even in the presence of rapamycin. This result confirms that autophagy plays an important protective role in damaged podocytes.

## Methods

### Ethics Statement

This study was approved by the Ethics Committee of The General Hospital of the People's Liberation Army (Permit Number: 2011-X3-50) with animal care performed strictly according to established institutional guidelines. All surgery was performed under pentobarbital anesthesia, and all efforts were made to minimize suffering.

### Experimental animals and induction of the PHN rat model

Male Sprague-Dawley (SD) rats were purchased from Vital River Laboratories and maintained under specific pathogen-free conditions: 22±1°C, 40% humidity, 12:12-h light/dark cycle, five males per cage, and free access to food and water. The initial body weight of the rats was 180 to 200 g.

Fx1A was isolated from SD rat kidneys by sieving and ultracentrifugation as described previously [24]. Rabbit anti-Fx1A was prepared by Beijing Biosynthesis Biotechnology Co. Ltd. (Beijing, China). Rats were injected with anti-Fx1A serum (1 mL/100 g body wt; antibody titer: 1:250) into the caudal vein, followed by another injection after 1 h. The control rats were injected with the same volume of normal saline. The rats were sacrificed 1, 7, 14, 21, and 28 days after injection for the following experiments (eight rats per group) and the renal cortex was isolated. Urine was collected 24 h prior to sacrificing.

### Reagents

PAN and rapamycin were purchased from Sigma-Aldrich (St. Louis, MO). The following primary antibodies were used: rabbit anti-synaptopodin and goat anti-synaptopodin (Santa Cruz Biotechnology, Santa Cruz, CA); anti-p-mTOR (Ser2448) (Cell Signaling, Danvers, MA); anti-p-70S6K (Thr389) (Cell Signaling); anti-p-4EBP1 (Ser65) (Cell Signaling); anti-p-ULK1 (Ser757) (Cell Signaling); rabbit anti-LC3 (Sigma-Aldrich) and moduse anti-LC3 (MBL Co. NaKa-ku Nagoya, Japan); and mouse anti-β-actin (Sigma-Aldrich). The following secondary antibodies were used: FITC and Cy3 conjugated IgG (Jackson ImmunoResearch Laboratories, PA, USA) and horseradish peroxidase conjugated IgG (Beyotime Shanghai, China).

### Isolation of rat glomeruli

Rats were anesthetized and kidneys were removed and washed with cold physiological saline. The renal capsule was stripped and the renal cortex was cut into pieces with scissors. Glomeruli were isolated by pressing minced kidneys through a 250 μm sieve, followed by passage through a 106 μm sieve and collected from the top of a 75 μm sieve. Tissues from the third sieve were sucked out and observed under the microscope, and when only the glomeruli and almost no tubular cells could be seen (glomerular purity >95%), the washes were complete and the collected tissues from this layer were moved into centrifuge tubes. After centrifugation, the supernatant was discarded and the isolated glomeruli precipitate was stored at −80°C.

### Cell culture and transfection

Conditionally immortalized mouse podocyte cells (MPCs) were kindly provided by Dr. Peter Mundel (University of Miami, Miami, FL, USA) and were cultured as previously described [25]. MPC cells were maintained in RPMI 1640 medium (Sigma-Aldrich) supplemented with 10% fetal calf serum (FCS; Life Technologies, Rockville, MD), 100 U/mL penicillin G, and 100 µg/mL streptomycin in a humidified 5% CO_2_ atmosphere. To propagate MPC cells, the culture medium was supplemented with 10 U/mL recombinant mouse γ-interferon (Pepro Tech EC Ltd., London, England) to enhance the expression of T-antigen, and the cells were cultured at 33°C (permissive conditions). Cells were shifted to 37°C in γ-interferon-free medium for 7 days (nonpermissive conditions) to induce differentiation. MPC cells between 7–14 d were used in all experiments. Cells were cultured in the absence or presence of PAN (50 μg/mL) in a time-dependent manner in RPMI-1640 medium that contained 100 U/mL penicillin/streptomycin supplemented with 5% fetal bovine serum at 37°C. For mTOR inhibition experiments, cells were cultured in the presence of rapamycin at the concentration of 200 ng/mL for 1 h before PAN incubation.

The jetPRIME™ (Polyplus-transfection Company) transfection reagent was used on differentiated MPCs for plasmid or siRNA transfection, and immunofluorescence analysis was applied for detecting transfection efficiency.

### Immunofluorescence

For indirect immunofluorescence (IF), the optimal cutting temperature (OCT)-embedded frozen renal cortex was cut into 4-µm slices and then fixed in 4% paraformaldehyde for 10 min. After washing with PBS, sections were blocked with 10% casein in deionized water for 30 min at room temperature and incubated with primary antibodies overnight at 4°C in a moisture chamber. Unbound antibodies were removed by washing with PBST. Secondary antibodies labeled with fluorescein were applied for 1 h at room temperature and washed as with the primary antibodies. MPCs were cultured to 70% confluence in special glass-bottom microwell dishes (MatTek Corporation, Ashland, MA, USA), then fixed with 4% paraformaldehyde and incubated with antibodies after permeabilizing with 0.2% Triton-X-100. All images were taken using a laser-scanning microscope (FV1000, Olympus, Tokyo, Japan).

### Western blot analysis

The glomeruli and cells were lysed in RIPA buffer (50 mM Tris-Cl [pH 7.6], 5 mM EDTA, 150 mM NaCl, 0.5% NP-40, and 0.5% Triton-X-100) containing 1 μg/mL leupeptin, aprotinin, and antipain; 1 mM sodium orthovanadate; and 0.5 mM phenylmethylsulfonyl fluoride. The protein concentration was measured using the Bradford assay. A total of 50 μg total protein was separated by 6–15% SDS-PAGE and then transferred to a membrane, which was blocked with 5% skim milk, probed with a primary antibody overnight at 4°C, and incubated with a horseradish peroxidase-conjugated secondary antibody.

### Statistical analysis

All data analyses were performed with SPSS 11.0 (SPSS Inc., Chicago, IL); data are expressed as means ±SD. Comparison among groups was conducted with ANOVA. *P*<0.05 was considered significant.

## Results

### Successful preparation of passive Heymann nephritis (PHN) rat model

**Figure 1:**
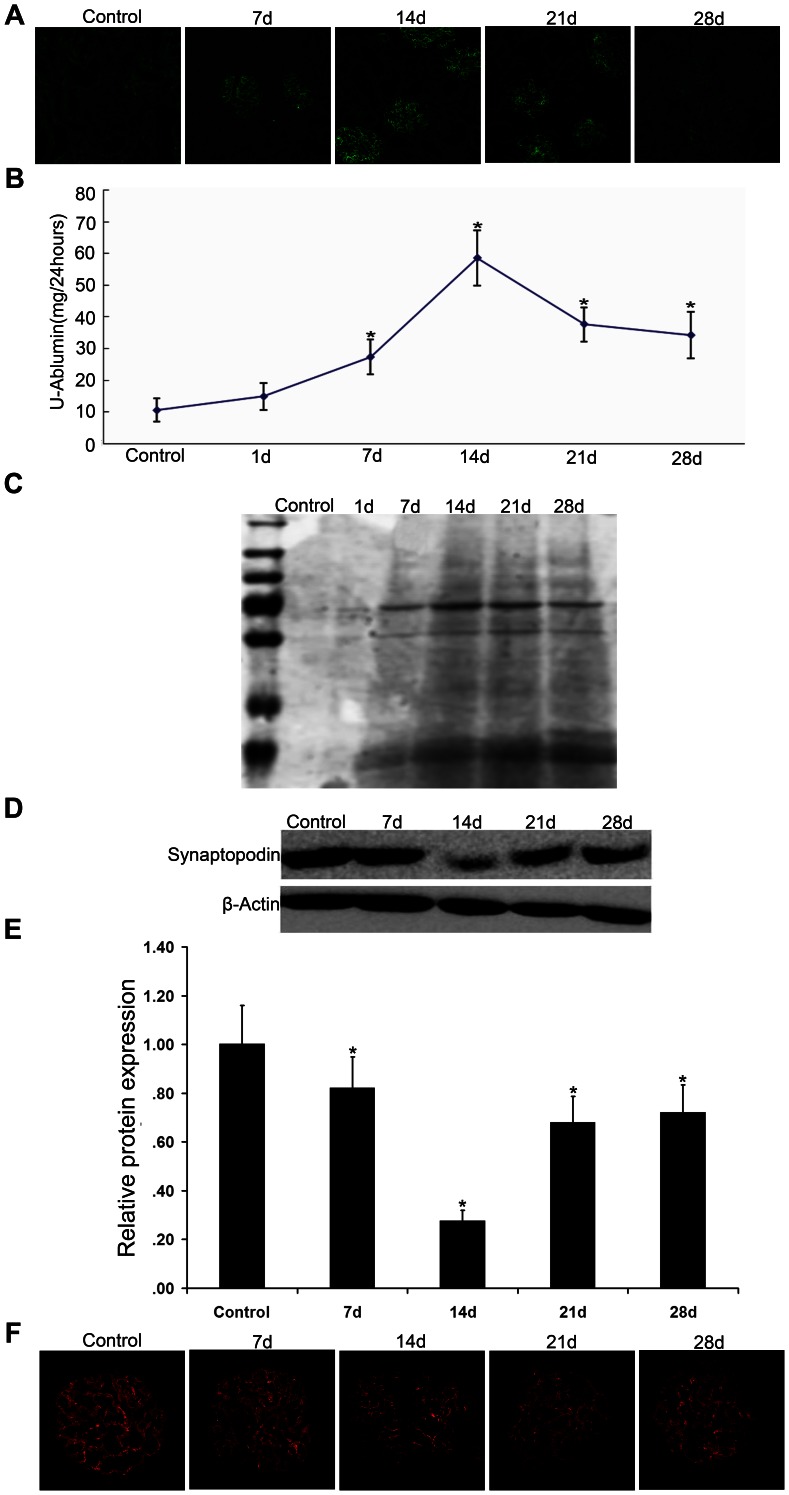
Preparation of passive Heymann nephritis rat model. (A).At day 1, 7, 14, 21, and 28 after antiserum injection, rats were sacrificed and the renal cortex was removed. Cryosections of the renal cortex were stained with goat anti-rat IgG-fluorescein isothiocyanate (FITC). Microscopic examination revealed that there were IgG depositions in the glomeruli of PHN rats compared to those of control rats. Depositions gradually increased over time, peaking on day 14. Magnification  =  × 400 (B). 24-hr urine was collected on days 0 (control), 1, 7, 14, 21, and 28, and analyzed for urinary albumin content using Coomassie Brilliant Blue G-250. (C). Electrophoresis followed by Coomassie blue staining. Each group urine sample was subjected to SDS-PAGE. Obvious albuminuria was apparent from day 7. (D-E).Western blot analysis of isolated glomerular protein on day 14 showed significantly reduced expression of synaptopodin in PHN rats compared to that in controls, *P<0.05 vs Control. (F).From immunofluorescent staining with goat anti-synaptopodin and confocal microscopic analysis of cryosections, we found that synaptopodin (red, a podocyte marker) expression in PHN rats was downregulated, and developed a fractured discontinuous-like distribution, compared to that observed in control rats. Magnification  = ×600.

On days 1, 7, 14, 21, and 28 after antiserum injection, the renal cortex was removed from sacrificed rats. In addition, urine specimens were collected 24 h prior to sacrifice. On modeling day 1, there were large numbers of IgG granular depositions in the glomeruli of PHN rats; deposition gradually increased and then decreased on day 28 (Figure 1A). Coomassie Brilliant Blue staining revealed that the rats had massive proteinuria at 7 d, which peaked at 14 d (Figure 1B and Figure 1C), indicating that the model was successfully established.

To verify that the podocytes in the PHN rat kidneys were injured, we examined the expression of synaptopodin, a podocyte protein marker. Western blot analysis of isolated glomerular protein showed that synaptopodin expression in PHN rats was downregulated on day 7 compared to control rats, and showed the largest decreased on day 14 (Figure 1D and Figure 1E). The timing of this expression profile was consistent with the peak of proteinuria. Immunofluorescence staining results revealed that PHN rats expressed significantly lower synaptopodin on day 14 than that expressed in the control group. Moreover, the fractured discontinuous-like distribution of synaptopodin (Figure 1F) indicated podocyte injury.

### Change in mTOR autophagy activity is related to podocyte injury in PHN rats

Under physiological conditions, podocytes maintain extremely low mTOR levels and high autophagy activity levels. When glomerular stress events eventually shift this balance toward increased mTOR expression and decreased autophagy activity, podocyte homeostasis is destroyed, leading to kidney diseases. Activation of mTOR leads to the phosphorylation of two downstream proteins, p70S6K and 4EBP1; therefore, the phosphorylation levels of these two proteins are frequently used as an indicator for mTOR activity [26], [27]. As a negative regulator of autophagy, activation of the mTOR pathway downregulates autophagy, destroys podocyte homeostasis, and injures podocytes. Therefore, we suspect that podocyte injury in PHN rats may be associated with changes in mTOR autophagy activity.

**Figure 2:**
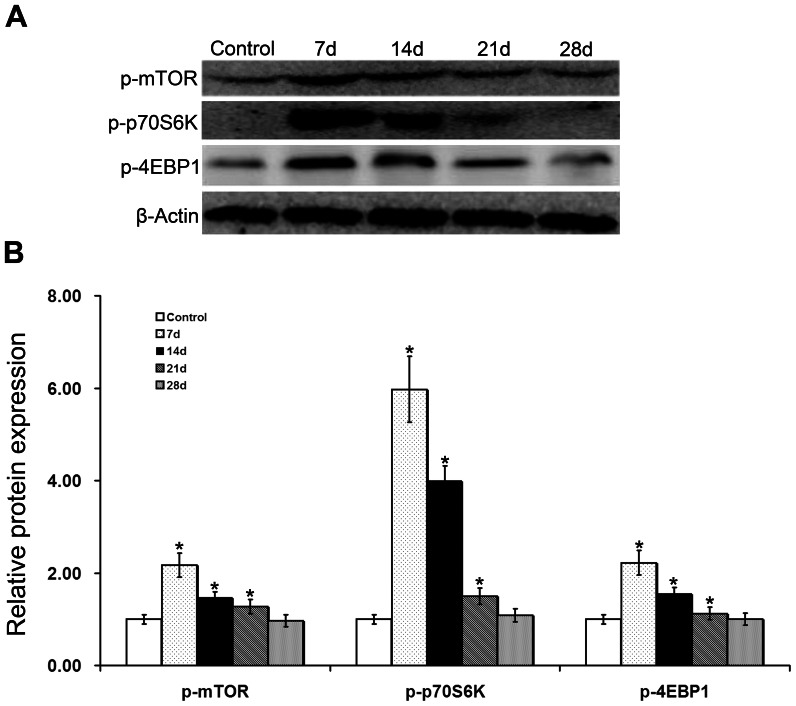
Activation of the mTOR signaling pathway in the glomeruli of PHN rats. Compared to control rats, the phosphorylation of mTOR, p70S6K, and 4EBP1 was enhanced in PHN rats on day 7 and continued to day 14, indicating that the mTOR signaling pathway was activated, *P<0.05 vs Control.

**Figure 3:**
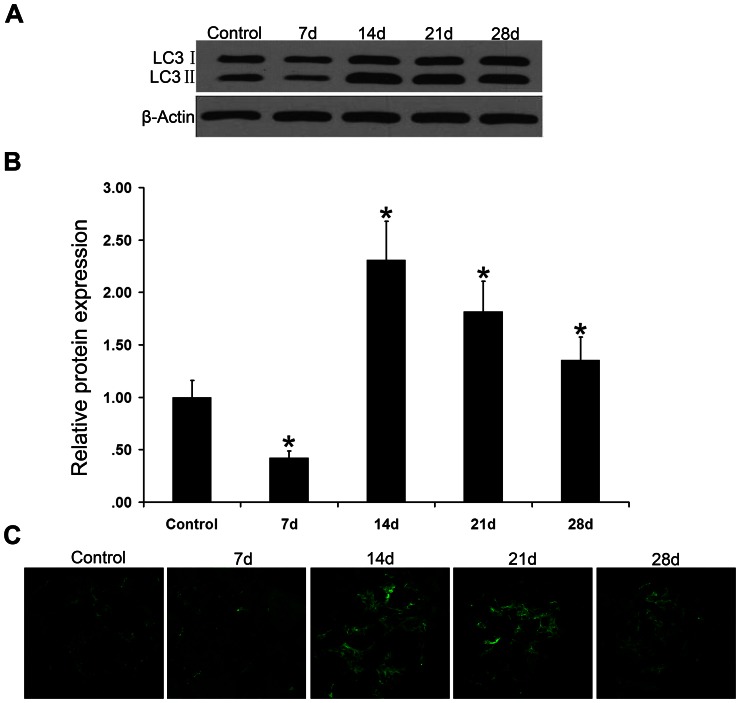
Autophagy level was first decreased and then increased in PHN rat glomeruli. (A–B).Western blot analysis of isolated glomerular protein confirmed the downregulation of LC3 II (an autophagy marker) in the glomeruli from PHN rats on day 7 compared to control rats, indicating decreased autophagy level. From day 14, the expression of LC3 II was upregulated, indicating increased autophagy level, *P<0.05 vs Control.(C).Confocal microscopic analysis of renal cortex cryosections from PHN rats and controls confirmed the expression of LC3 (green, an autophagy marker), indicating that the autophagy level first decreased and then increased in PHN rat glomeruli compared to that in control rats. Magnification  = ×600.

Using Western blot analysis, we measured the expression levels of mTOR pathway-associated proteins in the glomeruli from control and PHN rats. Compared to phosphorylation levels in control rats, those of mTOR, p70S6K, and 4EBP1 were significantly upregulated in PHN rat glomeruli on day 7, which continued to day 14 (Figure 2), indicating that the mTOR pathway was activated in PHN rats. Moreover, the autophagy level was lower in PHN rats than in controls; autophagy activity was inhibited, the mTOR-autophagy balance was broken, and podocytes were injured. From day 14, the autophagy level increased, and this increase continued to day 28 (Figure 3). When mTOR activity decreased, podocyte injury was reduced, suggesting that podocyte injury might be associated with the change in mTOR autophagy activity.

### Podocyte injury and activation of the mTOR-ULK1 pathway

**Figure 4:**
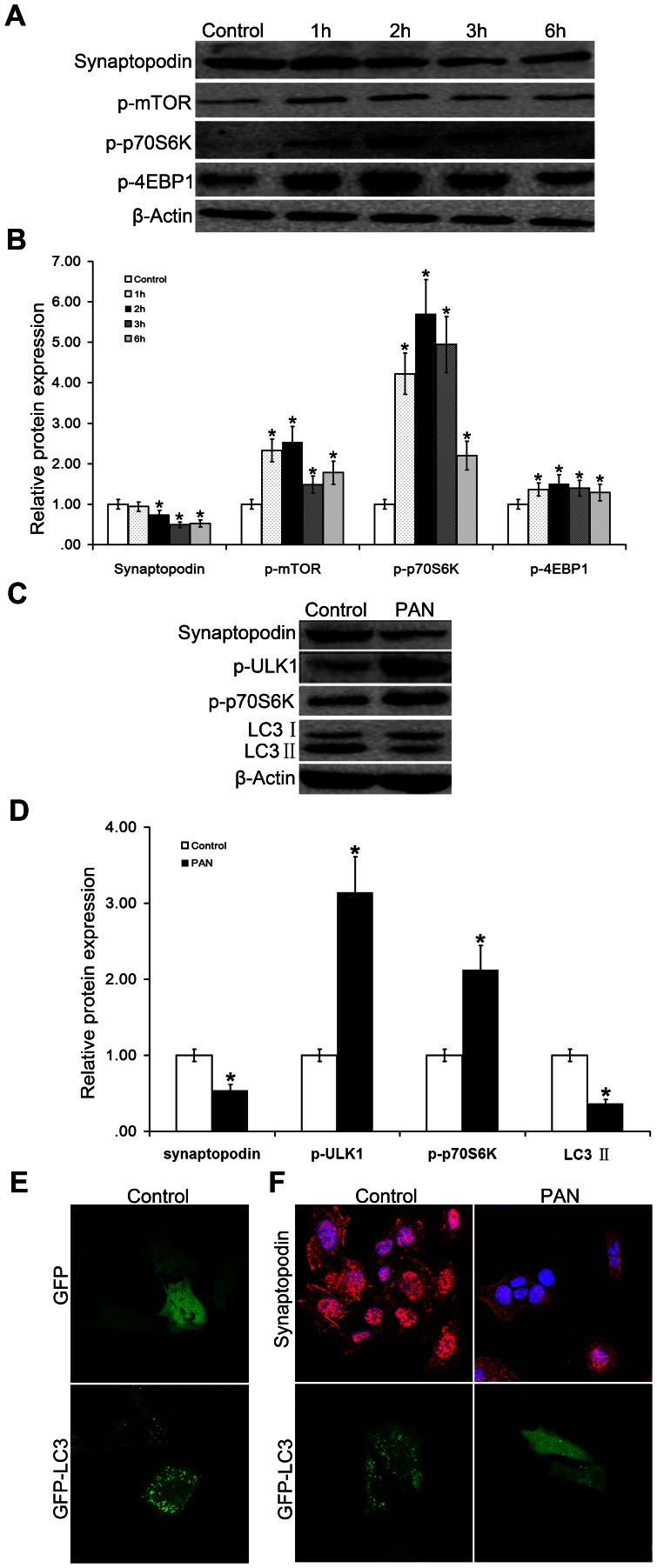
Podocyte injury and activation of the mTOR-ULK1 pathway. (A-B).Western blot analysis of cellular proteins confirmed that the most significant upregulated expression of mTOR signaling pathway-associated proteins was observed in 2 h puromycin amino nucleotide (PAN)-treated differentiated mouse podocyte cells (MPCs) compared to that in the control group and the other time points of treated cells, demonstrating the obvious activation of the mTOR-ULK pathway, *P<0.05 vs Control. (C, D, F).At this same time point, the expression of synaptopodin decreased and the filamentous expression (red fluorescence) was barely visible; therefore, we selected 2 h as the modeling time. PAN-treated podocytes showed an increase in ULK1 phosphorylation, a decrease in LC3 II expression, and a decrease in cellular GFP-LC3 granular expression (green fluorescence), indicating the inhibition of autophagy.(E).Compared to GFP-LC3 transfected cells, there was no granulation in GFP transfected cells. Magnification  = ×1200.

To examine the relationship between mTOR autophagy activity changes and podocyte injury, we established an *in vitro* podocyte injury model by treating conditionally immortalized mouse podocyte cells (MPCs) with the nephrotoxic drug PAN [28], [29] in a time-dependent manner. As shown in Figure 4A and Figure 4B, the phosphorylation levels of mTOR, p70S6K, and 4EBP1 were significantly increased in the 2 h PAN-treated differentiated MPCs compared to controls, indicating the activation of the mTOR pathway. In addition, synaptopodin (a podocyte marker) was downregulated and cells were injured. These results suggest that the model was successfully prepared, and we used 2 h PAN-treated cells in all subsequent experiments.

After establishing the model, we found that the cellular autophagy level was lower in PAN-treated podocytes than in control cells. Moreover, the Ser757 phosphorylation of ULK1 (Figure 4C and Figure 4D), a member of the initial complex of autophagy, was enhanced, autophagy was inhibited, and podocytes were injured. In addition, PAN stimulated a decrease in LC3-II protein and synaptopodin (Figure 4C and Figure 4D). The autophagy level could be detected as granular accumulations of GFP-LC3 [30]. As a control, we transfected differentiated MPCs with the plasmid that expressed GFP only, which there only showed a diffuse distribution of green fluorescence while we could observe granulation in GFP-LC3 transfected cells (Figure 4E). Transfected cells demonstrated a transformation from granular to dispersion after adding PAN (Figure 4F), indicating the inhibition of autophagy by PAN. From the results of immunofluorescence staining, we found that the overall state of PAN-treated podocytes was poor compared to the control group, the expression of synaptopodin (red fluorescence) was reduced, and filamentous expression was barely detectable. These results suggest that, through the mTOR-ULK1 pathway, mTOR downregulated the autophagy level, destroyed podocyte homeostasis, and injured podocytes.

### Rapamycin reduces podocyte injury caused by mTOR-ULK1 pathway activation

**Figure 5:**
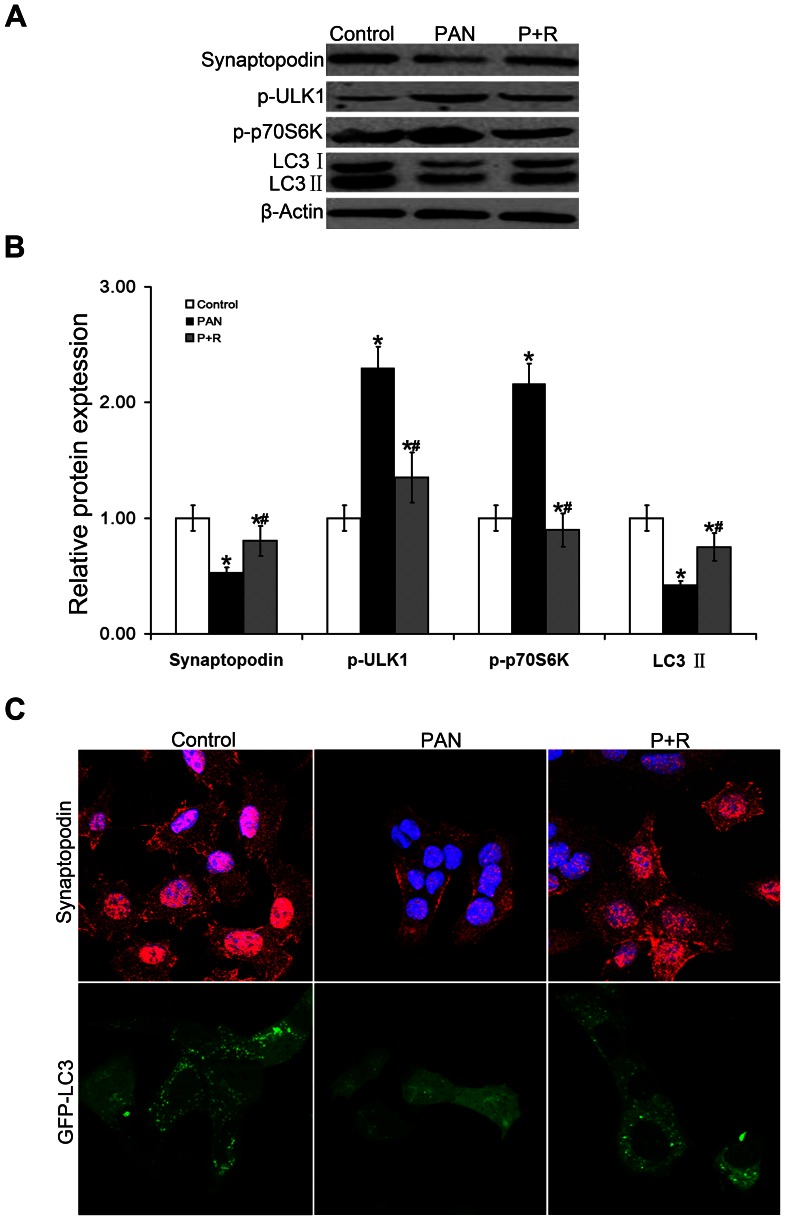
Rapamycin reduced podocyte injury by inhibiting the mTOR-ULK1 signaling pathway. (A–B).Western blot analysis of cellular proteins showed recovery of synaptopodin and LC3 II expression, a decrease in mTOR activity, and a decrease in ULK1 phosphorylation in rapamycin-pretreated cells (P+R) compared to PAN-treated cells in the absence of rapamycin (PAN), *P<0.05 vs Control, ^#^ *P*<0.05 vs PAN. (C).P+R podocytes appeared healthy with relatively round nuclei, enhanced synaptopodin expression (red fluorescence), and increased cellular GFP-LC3 granular expression (green fluorescence) compared to PAN cells. Magnification  = ×1200.

Previous experiments have found that activation of the mTOR-ULK1 pathway destroys podocyte homeostasis and induces podocyte injury. Furthermore, inhibiting the activation of the mTOR-ULK1 pathway reduces podocyte injury. To verify this conjecture, we pretreated differentiated MPCs with the classic mTOR pathway inhibitor, rapamycin. Both Western blot (Figure 5A and Figure 5B) and immunofluorescence (Figure 5C) showed that synaptopodin expression was significantly higher in the rapamycin-pretreated PAN-stimulated cells than in the untreated PAN-stimulated group. In addition, the rapamycin-treated cells had a higher autophagy level and a decreased phosphorylation level of ULK1 and mTOR signaling pathway-associated proteins. These results suggest that the activation of the mTOR-ULK1 pathway is involved in the process of podocyte injury. Inhibition of the mTOR-ULK1 pathway reduces PAN-induced podocyte injury, suggesting that rapamycin may function by upregulating autophagy level.

### Protective effect of autophagy on podocytes

**Figure 6:**
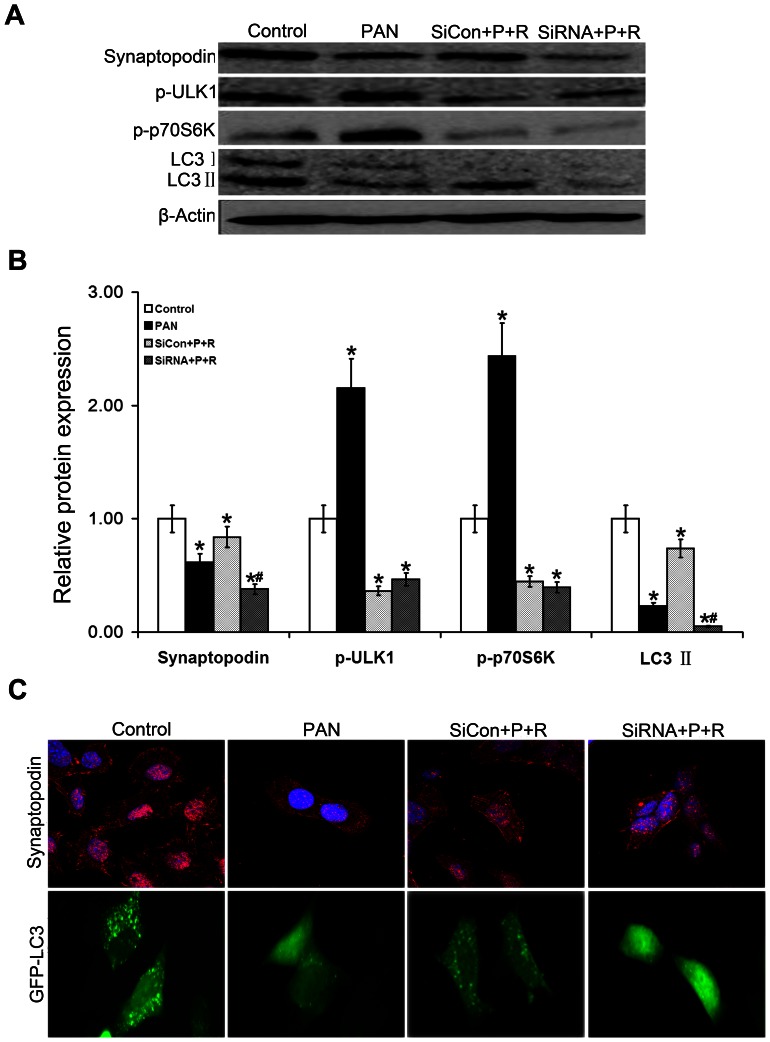
Aggravation of podocyte injury upon autophagy inhibition. (A–B).The Western blot results confirmed that the expression of synaptopodin and LC3 II was lower in ATG7 (a key protein of autophagy) RNAi-mediated knock down differentiated MPCs (siRNA+R+P) than in the siRNA negative control group (siCon+R+P). Both groups were pretreated with rapamycin and treated with PAN,*P<0.05 vs Control, ^#^ *P*<0.05 vs siCon+R+P. (C).In the presence of siRNA interference, GFP-LC3 granular expression (green fluorescence) nearly disappeared and cells appeared unhealthy with long and narrow nuclei. Synaptopodin expression (red fluorescence) was significantly downregulated. Magnification  = ×1200.

Earlier studies have shown that the autophagy level is much higher in podocytes than in other renal cells, suggesting that autophagy plays an important role in maintaining podocyte homeostasis. Autophagy helps podocytes cope with adverse environmental stress and allows them to recover from injury. To prove the protective effect of autophagy in podocytes, we used siRNA interference of ATG7 (a key protein of autophagy) to interfere with autophagy. After interfering with autophagy, we found that the expression of synaptopodin was still subject to inhibition (Figure 6) even when differentiated MPCs were pretreated with rapamycin to inhibit the mTOR pathway. This result suggests that autophagy has a protective effect on injured podocytes, plays a part in its repair process, and is involved in the maintenance of podocyte homeostasis.

## Discussion

Autophagy as a cellular protective mechanism has received extensive attention from many scholars, and mTOR as a major negative regulator of autophagy has been paid increasing attention. In the present study, we first found that podocyte injury in PHN rats was associated with changes in mTOR autophagy activity. At the same time, the changes in autophagy levels and mTOR activation were negatively correlated in injured podocytes, and the mTOR–autophagy balance of low-level mTOR and high-level autophagy was uncoupled, consistent with previous reports. *In vitro* studies showed that mTOR inhibited autophagy by phosphorylating ULK1, a member of the autophagy initiation associated complex ULK1-ATG13-FIP200. This inhibition of autophagy disrupted podocyte homeostasis and damaged podocytes, however, treatment with rapamycin increased the level of autophagy and reduced podocyte injury by inhibiting the mTOR-ULK1 pathway. Moreover, after treating differentiated MPCs with siRNA that knocked out the expression of ATG7, a key protein of autophagy, rapamycin was not effective in its role. This result strongly suggests that autophagy plays an important role in maintaining podocyte homeostasis and has a protective effect on damaged podocytes.

Ken Inoki et al. [27] proposed that abnormal mTOR activation in podocytes involved a variety of molecular reactions, including the mislocalization of slit diaphragm proteins and the induction of an epithelial-mesenchymal transition-like phenotypic switch with enhanced ER stress in podocytes. Their study found that the abnormal activation of mTOR played a key role in podocyte injury and proteinuria found in human and rat diabetic nephropathy. Noriko Ito et al. [31] demonstrated that mTOR activation perturbed the regulatory system of energy metabolism primarily by promoting energy consumption and inducing the unfolded protein response (UPR), which underlied proteinuria in MCD. In our study we found that the mTOR pathway was activated at first, then the activation lasted for a certain time, and this tended to decrease to baseline level at last. These results are consistent with those of previous studies that showed that activation of the mTOR signaling pathway leaded to podocyte injury and a decrease in skeleton-associated protein synaptopodin expression. Our results indicate that podocyte injury involves the activation of the mTOR-ULK1 pathway and inhibition of autophagy; disruption of the mTOR–autophagy balance destroys podocyte homeostasis.

Because mTOR integrates signals that are emitted by growth factors, amino acids, glucose, and energy status, it plays a central role in protein synthesis and the negative regulation of autophagy [32], [33], [34], [35], [36]. The mTOR complex suppresses autophagy via phosphorylation and inactivation of ULK1, an initiator of autophagosome formation. On the other hand, mTOR can regulate autophagy by phosphorylating 4EBP1 and p70S6K to regulate protein synthesis and cell growth. Our results showed that when mTOR was activated, the p-ULK1 (Ser757) level was significantly increased and at this time, ULK1 could not phosphorylate Atg13 and FIP200 to trigger autophagy because it lost its kinase activity. Thus, mTOR broke the mTOR–autophagy balance, allowing podocyte injury by inhibiting autophagy. After pretreating cells with rapamycin, a classical mTOR inhibitor, we found that the mTOR pathway was inhibited, thereby increasing the autophagy level, recovering the mTOR–autophagy balance and reducing podocyte injury. When autophagy was inhibited using siRNA, the mTOR–autophagy balance could not recover and the inhibition of the mTOR pathway could not reduce podocyte injury. These results convincingly indicate that autophagy plays an important role in podocytes.

The results of this study confirm that autophagy plays an important role in maintaining podocyte homeostasis and protecting damaged podocytes. We showed, for the first time in podocytes, that mTOR inhibited autophagy by regulating the phosphorylation level of ULK1 and thus undermined the balance of the mTOR–autophagy balance to allow podocyte injury. Rapamycin reduced podocyte injury by inhibiting the mTOR-ULK1 pathway to increase the autophagy level. This discovery provides a potential new target for future treatment of kidney diseases with podocyte injury.
